# CD133+CD24+ Renal Tubular Progenitor Cells Drive Hypoxic Injury Recovery via Hypoxia-Inducible Factor-1A and Epidermal Growth Factor Receptor Expression

**DOI:** 10.3390/ijms26062472

**Published:** 2025-03-10

**Authors:** Sarmad Al-Marsoummi, Sonalika Singhal, Scott H. Garrett, Seema Somji, Donald A. Sens, Sandeep K. Singhal

**Affiliations:** 1 Department of Pathology, School of Medicine and Health Sciences, University of North Dakota, Grand Forks, ND 58202, USA; sarmad.al.marsoummi@und.edu (S.A.-M.); sonalika.singhal@und.edu (S.S.);; 2 Bioinformatics Division Adjunct Faculty, Pathology and Cell Biology, Columbia University Medical Centre, New York, NY 10032, USA

**Keywords:** HRTPT, kidney progenitor, hypoxia, RNA sequencing

## Abstract

CD133+CD24+ renal tubular progenitor cells play a crucial role in the repair and regeneration of renal tubules after acute kidney injury. The aim of this study is to investigate the responses of the human renal tubular precursor TERT (HRTPT) CD133+CD24+ cells and human renal epithelial cell 24 TERT (HREC24T) CD133-CD24+ cells to hypoxic stress, as well as their gene expression profiles. Whole transcriptome sequencing and functional network analysis identified distinct molecular characteristics of HRTPT cells as they were enriched with hypoxia-inducible factor-1A (HIF1A), epidermal growth factor (EGF), and endothelin-1 (EDN1). Our in vitro experiments demonstrated that, under hypoxia (2.5% oxygen), HRTPT cells showed minimal cell death and a 100-fold increase in HIF1A protein levels. In contrast, HREC24T cells exhibited significant cell death and only a two-fold increase in HIF1A protein level. These results indicate that CD133+CD24+ renal tubular progenitor cells have enhanced survival mechanisms under hypoxic stress, enabling them to survive and proliferate to replace damaged tubular cells. This study provides novel insights into the protective role of CD133+CD24+ renal tubular progenitor cells in hypoxic renal injury and identifies their potential survival mechanisms.

## 1. Introduction

End-stage renal disease (ESRD) is a global health problem with increasing incidence and prevalence [1]. Renal dialysis and kidney transplants remain the only treatment options for ESRD [2]. However, the high cost and medical complications associated with dialysis and the difficulty in obtaining kidney transplants emphasize the urgent need for alternative therapeutic options. Kidney stem cells and regenerative medicine may provide promising alternatives for patients with chronic kidney disease.

The human kidney has the intrinsic capacity to repair itself after acute, non-extensive damage [3]. This repair process is orchestrated by intrarenal cells, not cells recruited from outside the kidney [4,5,6]. The mechanism by which the kidney repairs itself after acute injury—whether through the dedifferentiation of undamaged tubular cells or through the activation of resident scattered progenitor cells—remains a topic of debate. However, evidence supports the presence of a resident, scattered population of cells with progenitor characteristics that survive the injury and can undergo clonal proliferation after injury to replace the damaged tubular cells [5,7,8]. CD133+CD24+ cells in the human kidney have been shown to possess progenitor characteristics and the capacity to regenerate tubular cells, supporting kidney repair after acute damage [7,9,10]. In vivo studies have demonstrated the ability of CD133+CD24+ cells to repair damaged kidney tubules and promote recovery of kidney function when these cells are injected into a mouse with acute kidney injury [9]. While both induced pluripotent stem cells (iPSCs) and kidney progenitor cells hold promise as potential therapy for kidney damage [11], kidney progenitors are considered to have a higher potential for success due to the lower propensity for dysplasia, hypertrophy, or cancerous transformation compared to iPSCs [12]. Understanding the biology of kidney progenitor cells is essential for successfully applying these cells in clinical applications. However, our understanding of CD133+CD24+ kidney progenitor cells and their role in kidney repair remained limited due to the challenges in obtaining primary kidney tissue and the sparse presence of this rare cell population within the kidney.

Our laboratory was the first to identify that primary kidney cortical tissue and its immortalized counterpart, the RPTEC/TERT1 proximal tubular cell line, consist of heterogeneous populations of CD133+CD24+ and CD133-CD24+ cells [13]. Furthermore, our laboratory successfully isolated and established an immortalized CD133+CD24+ cell line that we identified as human renal tubular precursor TERT (HRTPT) cells [10,13,14,15,16]. This HRTPT cell line exhibits kidney progenitor characteristics, including nephrosphere formation and multipotential differentiation capacity [10,16]. Additionally, we isolated from the same tissue and characterized CD24+ cells lacking CD133 expression, which we identified as human renal epithelial cells 24 TERT (HREC24T). HREC24T cells have no progenitor characteristics; therefore, HREC24T cells were considered differentiated tubular cells [10,16]. Both HRTPT and HREC24T cells maintained consistent phenotypic and CD133/CD24 expression patterns across multiple passages [10,15]. This immortalized CD133+CD24+ (HRTPT) kidney cell line offers a consistent and unlimited supply of tubular progenitor cells to study the biology of kidney progenitor cells.

In this study, we aimed to identify how the transcriptome of the immortalized CD133+CD24+ (HRTPT) cell line is distinct from that of the CD133-CD24+ (HREC24T) cells and to define the transcriptomic signatures that characterize these two cell lines. This is crucial for uncovering the mechanisms that drive kidney repair and regeneration after injury.

This study used a rigorous methodological approach to investigate the molecular and signaling pathways that differentiate HRTPT cells from HREC24T cells. RNA sequencing was performed on RPTEC/TERT1, HRTPT, and HREC24T cell lines to identify gene expression profiles and the genetic markers unique to CD133+CD24+ (HRTPT) cells, as well as the key regulatory pathways that drive the functional differences between HRTPT renal tubular precursor cells and HREC24T non-progenitor cells.

Our analysis revealed that HRTPT CD133+CD24+ progenitor cells are enriched with key genes involved in the hypoxia response and epidermal growth factor (EGF) signaling pathways. In vitro experiments demonstrated that HRTPT cells are more resistant to hypoxic injury than HREC24T and RPTEC/TERT1 cells, with significantly higher upregulation of HIF1A and EGFR in HRTPT cells under hypoxic conditions.

## 2. Results

### 2.1. Immortalized HRTPT Kidney Progenitor Cells Express PROM1 (CD133) and CD24

**Figure 1:**
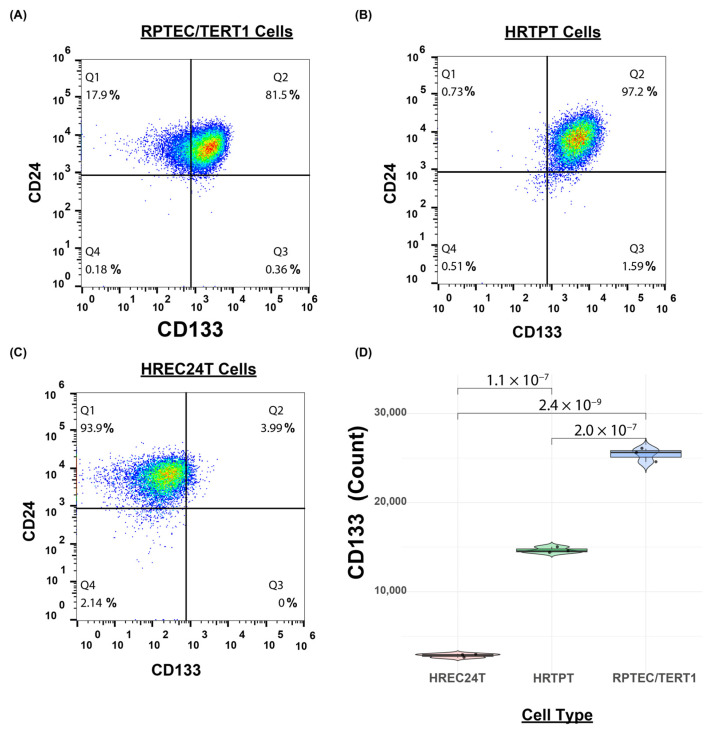
HRTPT cells express CD133 and CD24, and HREC24T cells express CD24. Flow cytometry analysis of PROM1 (CD133) and CD24 expression in (**A**) RPTEC/TERT1 cells, (**B**) HRTPT cells, and (**C**) HREC24T cells, showing that HRTPT cells express both CD133 and CD24; in contrast, HREC24T cells express only CD24. (**D**) A box plot of transcriptome analysis illustrating the expression levels of CD133 in RPTEC/TERT1, HRTPT, and HREC24T cells.

In our previous work, we successfully isolated and characterized two distinct cell lines from the parent RPTEC/TERT1 cells: HRTPT (CD133+CD24+) renal tubular progenitor cells and HREC24T (CD133-CD24+) non-progenitor cells [10,15]. Flow cytometry analysis confirmed that HRTPT cells express PROM1 (CD133) and CD24, while HREC24T non-progenitor cells express CD24 only (Figure 1A–C). Transcriptome analysis of fully confluent RPTEC/TERT1, HRTPT, and HREC24T cells demonstrated high PROM1 (CD133) expression in the parental RPTEC/TERT1 and HRTPT cells, but low CD133 expression in the HREC24T cells (Figure 1D).

### 2.2. Global Gene Expression Analysis of HRTPT, HREC24T, and RPTEC/TERT1 Cells

**Figure 2:**
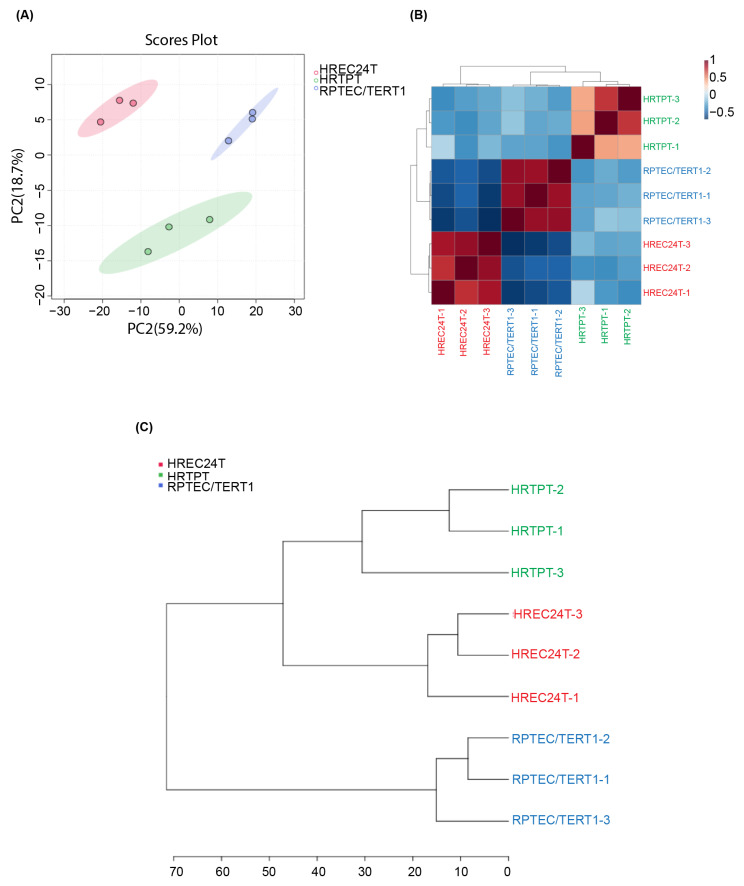
Transcriptome analysis of HRTPT, HREC24T, and RPTEC/TERT1 cells. (**A**) Principal component analysis (PCA) plot illustrating the clustering of HRTPT, HREC24T, and RPTEC/TERT1 triplicate samples based on the similarities in gene expression patterns. The two PCA components (PC1 and PC2) of the gene expression profile, along with the overall variance among the samples, are presented. Each dot represents a sample and is color-coded according to the cell line (green = HRTPT cells, red = HREC24T cells, and blue = RPTEC/TERT1 cells). (**B**) Hierarchical cluster analysis and heatmap illustrating the correlation among triplicate samples of HRTPT, HREC24T, and RPTEC/TERT1 cells. (**C**) A dendrogram illustrating the closely related samples of HRTPT, HREC24T, and RPTEC/TERT1 cells.

Whole transcriptome sequencing was performed on HRTPT, HREC24T, and RPTEC/TERT1 cells to elucidate gene expression patterns, followed by a comprehensive bioinformatic analysis. Principal component analysis (PCA) of triplicate samples from each cell line demonstrated distinct separation between HRTPT, HREC24T, and RPTEC/TERT1 cells, with the first principal component explaining 59.2% of the variance and the second 18.7% (Figure 2A). Hierarchical clustering further corroborated these observations and demonstrated a tight clustering of the triplicate samples within each cell type. This consistency indicated each group’s distinct gene expression profiles and highlighted significant differences among the three cell lines (Figure 2B,C).

**Figure 3:**
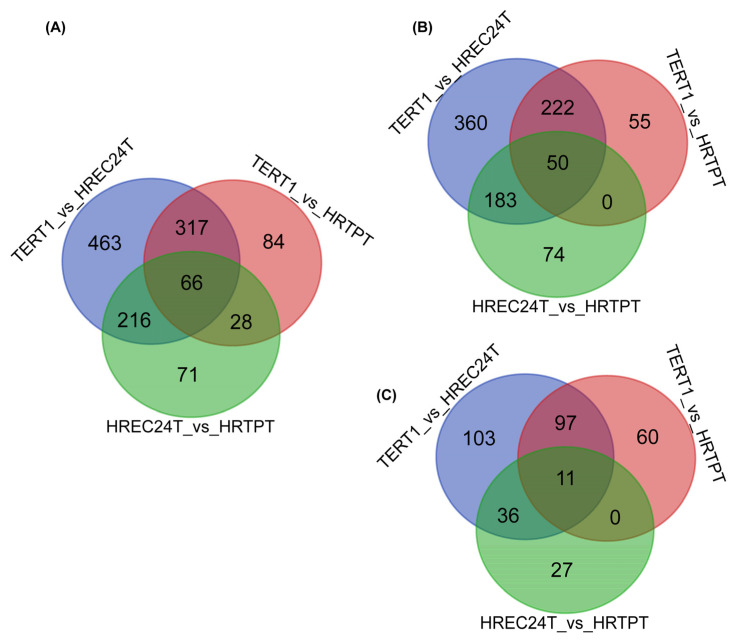
Differentially expressed genes in HRTPT, HREC24T, and RPTEC/TERT1 cells. Venn diagrams of the shared differentially expressed genes (DEGs) among HRTPT, HREC24T, and RPTEC/TERT1 cells with an adjusted *p*-value (*p*-adj) < 0.05 and an absolute value of log-fold change of ≥1.0. (**A**) Venn diagram of all downregulated and upregulated DEGs; (**B**) Venn diagram of only the upregulated DEGs; and (**C**) Venn diagram of only the downregulated DEGs.

A comprehensive analysis of differentially expressed genes (DEGs) was conducted to identify the significant DEGs among RPTEC/TERT1, HRTPT, and HREC24T cell samples. A total of 7679 DEGs were identified with adjusted *p*-values (*p*-adj) < 0.05. To refine this list, we identified DEGs with a *p*-adj < 0.05 and a log-fold change of ≥1.0, which resulted in a total of 1245 significant genes. When we compared HRTPT cells to RPTEC/TERT1 cells, 495 DEGs were identified (Supplemental Table S1), with 327 genes upregulated and 168 downregulated (Figure 3A–C). When the HREC24T cell line was compared to the RPTEC/TERT1 cell line, 1062 significant DEGs were identified (see Supplemental Table S2), with 815 upregulated and 247 downregulated genes (Figure 3A–C). Comparing the HRTPT cell line versus the HREC24T cell line resulted in 381 significant DEGs (Supplemental Table S3), with 307 upregulated and 74 downregulated genes (Figure 3A–C).

**Figure 4:**
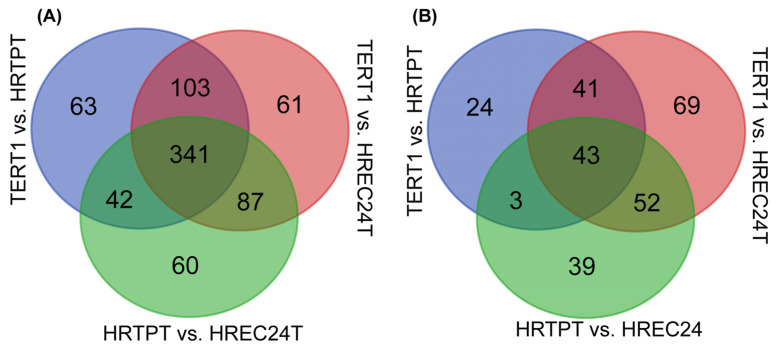
Functional pathways shared among HRTPT, HREC24T, and RPTEC/TERT1. Enrichment analysis of functional pathways using IPA based on the selected list of differentially expressed genes (DEGs): (**A**) DEGs with an adjusted *p*-value (*p*-adj) < 0.05. (**B**) DEGs with *p*-adj < 0.05 and an absolute log-fold change ≥1.0 value.

Pathway enrichment analysis was performed using the significant DEGs, and 341 enriched functional pathways were identified among the three cell lines (Figure 4A). After narrowing the analysis to DEGs with *p*-adj < 0.05 and a log-fold change of ≥1.0, the number of shared enriched pathways was reduced to 43 only (Figure 4B). Additionally, 39 pathways were uniquely enriched in HRTPT cells, and 69 pathways were unique to HREC24T cells. These findings indicated the unique molecular mechanisms and functional pathway differences between the HRTPT progenitor and HREC24T non-progenitor cell lines.

### 2.3. Ingenuity Pathway Analysis Indicates That HRTPT Cells Are Enriched with Hypoxia Response Elements

**Figure 5:**
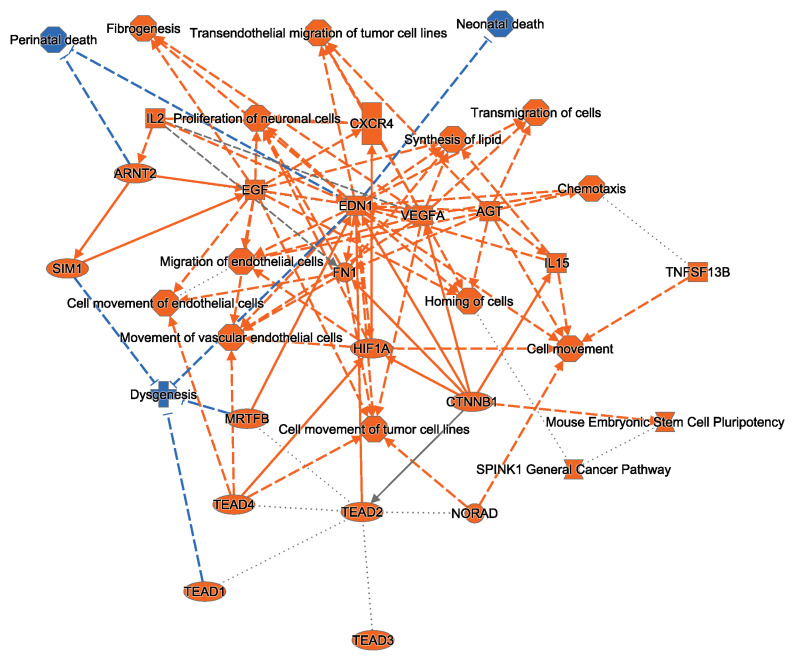
Ingenuity Pathway Analysis (IPA) of the differentially expressed genes in HRTPT Cells. Ingenuity Pathway Analysis (IPA) in HRTPT cells showing significant enrichment in the hypoxia-inducible factor 1-alpha (HIF1A) and epidermal growth factor (EGF) signaling pathways. The differential expression was analyzed by comparing HRTPT cells with HREC24T cells as control (orange color indicates pathway activation, while blue color indicates pathway inhibition, solid line indicates direct interaction, and dashed line indicates indirect interaction).

To elucidate the enriched molecular mechanisms distinguishing HRTPT cells and HREC24T cells, we performed Ingenuity Pathway Analysis (IPA) using the differentially expressed genes (DEGs) identified from global gene expression analysis. The IPA core analysis revealed a significant enrichment of several canonical pathways (Figure 5). In particular, the top molecular network constructed from DEGs showed that HRTPT progenitor cells were enriched in pathways related to human and mouse embryonic stem cell pluripotency and transcriptional regulatory networks important in controlling stem cell function (Supplemental Table S4).

Further analysis of upstream regulators indicated that HRTPT cells were enriched in pathways associated with the transcriptional regulator hypoxia-inducible factor-1A (HIF1A) and the epidermal growth factor (EGF) cytokine, with endothelin-1 (EDN1) acting as a central connecting node. These findings suggest that CD133+CD24+ HRTPT progenitor cells play a pivotal role in mediating the response of the kidney to hypoxic injury, potentially through the regulation of hypoxia-inducible factors and epidermal growth factor signaling.

### 2.4. HRTPT CD133+CD24+ Kidney Progenitor Cells Are Resistant to Hypoxic Cell Death

To investigate if the identified pathways in our bioinformatic analysis (that showed HRTPT cells were enriched in functional pathways related to hypoxia response and epidermal growth factor) correlate with hypoxic stress response, we cultured fully confluent HRTPT (CD133+CD24+) cells and HREC24T (CD133-CD24+) cells under hypoxic conditions (2.5% oxygen) for 24, 48, and 72 h to investigate their response to hypoxia.

**Figure 6:**
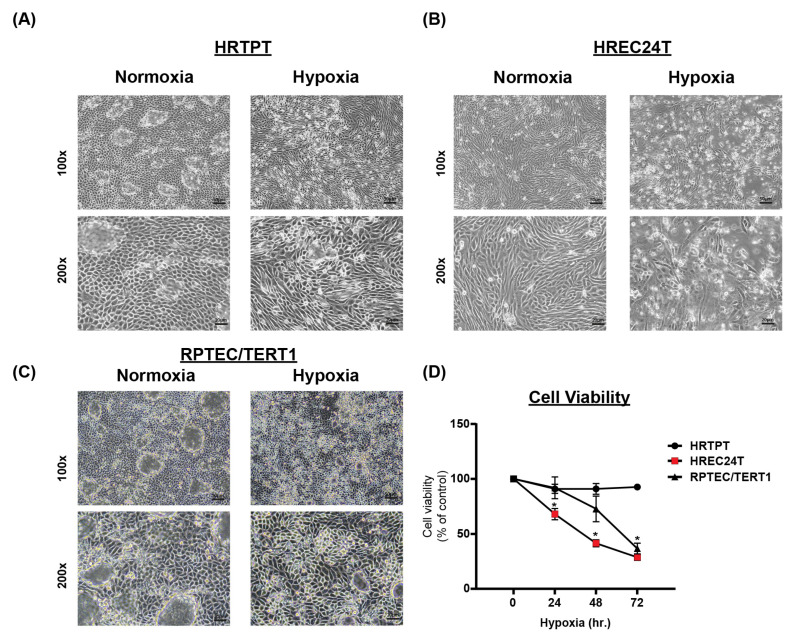
(**A**–**C**) Microscopic images of HRTPT, HREC24T, and RPTEC/TERT1 cells cultured under hypoxia (2.5% oxygen) or normoxia (as control) for 48 h (scale bars: 50 µm for 100× magnification and 20 µm for 200× magnification). (**D**) Cell viability analysis by crystal violet staining of HRTPT, HREC24T, and RPTEC/TERT1 cells cultured for 72 h under hypoxia (2.5% oxygen), with cells cultured under normoxia as control. Data are expressed as mean ± SEM. * *p* < 0.05.

Morphologically, HRTPT cells showed no signs of cell death and maintained normal dome formation under hypoxic stress (Figure 6A). In contrast, HREC24T cells showed significant cell death under the same conditions (Figure 6B). RPTEC/TERT1 cells also showed signs of cell death when exposed to hypoxia (Figure 6C); however, cell death was less prominent than that observed in HREC24T cells.

Cell viability analysis using crystal violet staining revealed a significant reduction in the viability of HREC24T cells under hypoxia at all time points (24, 48, and 72 h). In contrast, HRTPT progenitor cells showed no significant reduction in cell viability under hypoxic stress (Figure 6D). Similarly, RPTEC/TERT1 cells exhibited no significant decrease in cell viability after 24 h of hypoxic exposure; however, a marked reduction was observed after 48 and 72 h of hypoxia. Nevertheless, the decline in RPTEC/TERT1 cell viability was less pronounced than the cell death observed in HREC24T cells, suggesting a differential response to hypoxic stress between HRTPT and HREC24T cells.

### 2.5. Hypoxia Upregulates HIF1A and EGFR Expression in HRTPT Progenitor Cells

To validate the results from our transcriptomic and functional pathway analyses and to determine whether the identified pathways contribute to the increased resistance of HRTPT cells to hypoxic stress, we cultured HRTPT and HREC24T cells under hypoxic conditions (2.5% oxygen) for 48 h, followed by protein and mRNA analyses.

**Figure 7:**
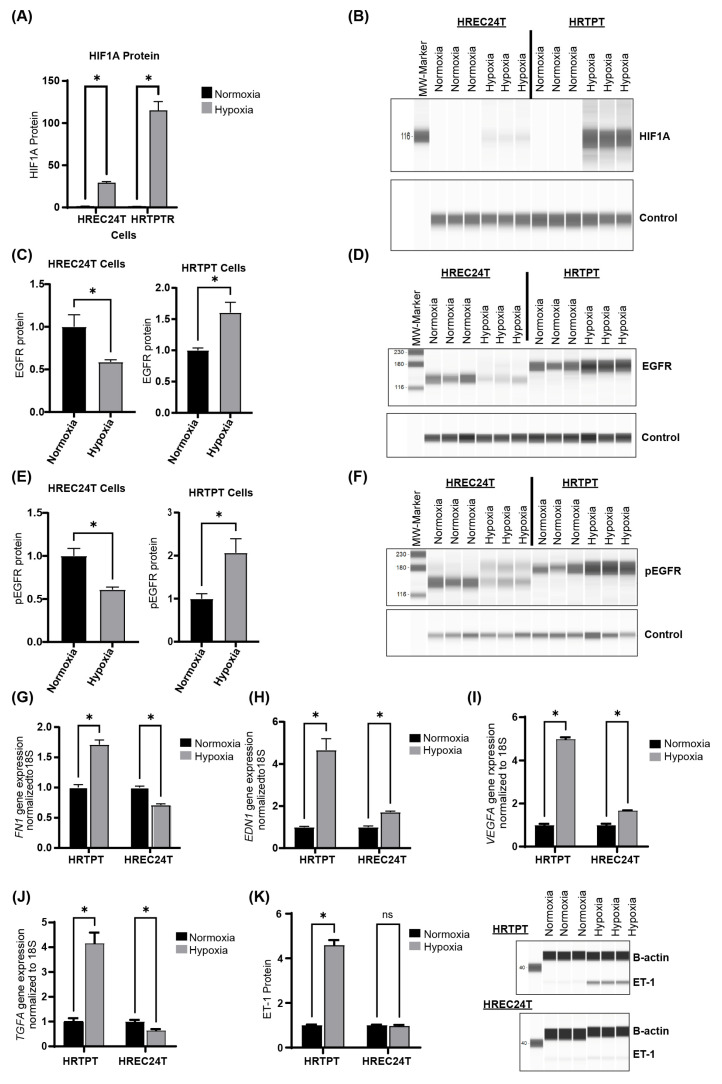
Effects of hypoxia on HRTPT and HREC24T cells. Protein analysis by Simple Western™ of (**A**,**B**) HIF1A, (**C**,**D**) EGFR, and (**E**,**F**) phosphorylated EGFR (pEGFR) in HRTPT and HREC24T cells cultured for 48 h under hypoxia (2.5% oxygen). Cells cultured under normoxia were used as controls. Gene expression analysis by RT-qPCR of (**G**) *FN1*, (**H**) *EDN1*, (**I**) *VEGFA*, and (**J**) *TGFA* in HRTPT and HREC24T cells cultured for 48 h under hypoxia (2.5% oxygen). Cells cultured under normoxia used as controls. (**K**) Protein levels analysis by Simple Western ™ of endothelin-1 (ET-1) in HRTPT and HREC24T cells cultured for 48 h under hypoxia (2.5% oxygen). Cells cultured under normoxia were used as controls (Student’s *t*-test, ns: not significant * *p* < 0.05, *n* = 3). Data are presented as mean ± SEM.

Both HRTPT and HREC24T cells cultured under hypoxic conditions exhibited a significant increase in HIF1A protein levels. However, the upregulation of HIF1A protein in HRTPT cells was 100-fold greater than in HREC24T cells (Figure 7A,B). Additionally, hypoxia significantly increased the protein levels of total epidermal growth factor receptor (EGFR) and phosphorylated EGFR (pEGFR) in HRTPT progenitor cells. In contrast, HREC24T cells showed a significant decrease in EGFR and pEGFR expression under hypoxic stress (Figure 7C–F). Furthermore, hypoxia led to a significant increase in the expression of endothelin-1 (EDN1) and fibronectin-1 (FN1) in HRTPT progenitor cells (Figure 7G,H), with EDN1 and FN1 upregulation being four times greater in HRTPT progenitor cells compared to HREC24T non-progenitor cells. Hypoxia also significantly increased the expression of HIF1A target genes, vascular endothelial growth factor A (VEGFA), and transforming growth factor alpha (TGFA), with VEGFA and TGFA expression in HRTPT cells being four times higher than in HREC24T cells (Figure 7I,J). Moreover, endothelin-1 protein (ET-1) levels significantly increased in HRTPT cells under hypoxia, whereas no significant increase was observed in HREC24T cells (Figure 7K).

### 2.6. HREC24T and HK-2 Cells Have Comparable Responses to Hypoxic Stress

We sought to compare the responses of HREC24T non-progenitor cells with those of the widely studied HK-2 proximal tubular cell line. Our previous research demonstrated that fewer than 5% of HK-2 cells co-express CD133 and CD24, while 95% express only CD24 [14]. To further investigate, we compared the transcriptomes of RPTEC/TERT1, HRTPT, and HREC24T cell lines with the previously published transcriptome of human renal proximal tubular epithelial cells (HK-2) from GSE168559. This analysis assessed the relationship between our cell lines and another commercially available immortalized proximal tubule epithelial cell line derived from normal adult human kidneys.

**Figure 8:**
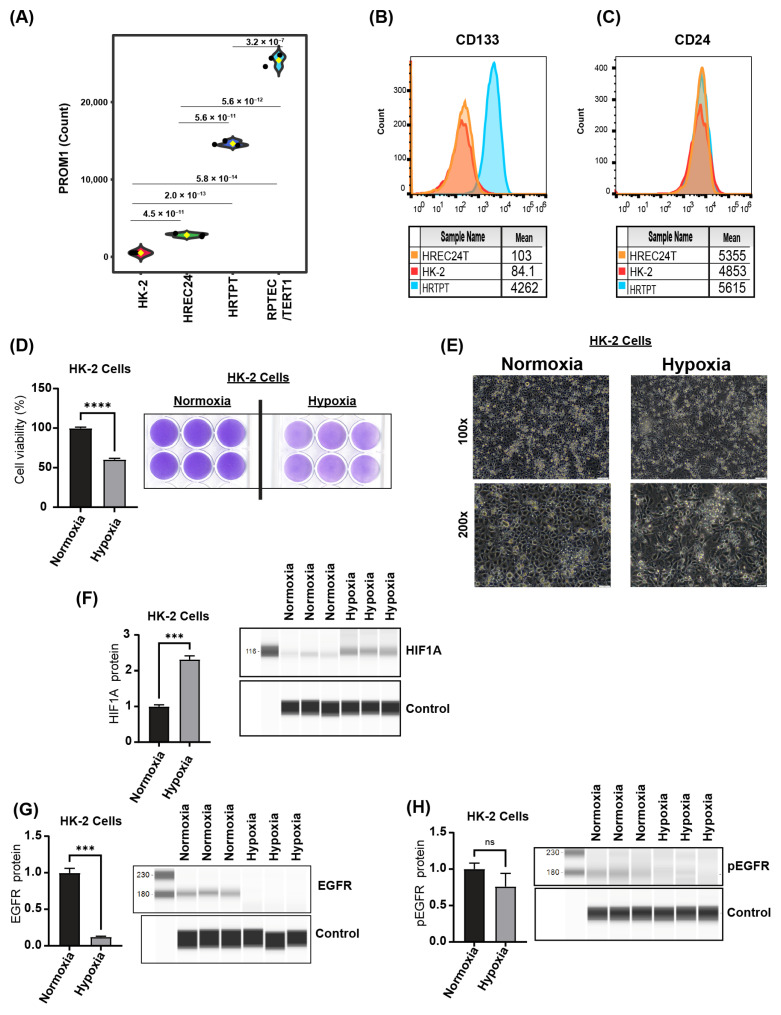
Effects of hypoxia on HK-2 cells. (**A**) Box plot of transcriptome analysis showing PROM1 (CD133) expression levels in RPTEC/TERT1, HRTPT, HK-2, and HREC24T cell lines. (**B**,**C**) Histograms showing CD133 and CD24 protein levels, analyzed by flow cytometry, in HRTPT, HREC24T, and HK-2 cells (mean = mean fluorescence intensity, orange color = HREC24T cells, red color = HK-2 cells, blue color = HRTPT cells). (**D**) Cell viability assessed by crystal violet labeling in HK-2 cells cultured under either hypoxia (2.5% oxygen) or normoxia for 48 h with a representative image of the crystal violet labeling. (**E**) Microscopic images of HK-2 cells cultured under hypoxia for 48 h or normoxia as control (scale bars: 50 µm for 100× magnification and 20 µm for 200× magnification). Protein level analysis by ProteinSimple™ of (**F**) HIF1A, (**G**) EGFR, and (**H**) pEGFR proteins in HK-2 cells cultured under hypoxia (2.5% oxygen) for 48 h. (*** *p* < 0.001, **** *p* < 0.0001, ns: not significant, Student’s *t*-test, *n* = 3). Data are expressed as mean ± SEM.

Transcriptome analysis revealed a clear distinction between the RPTEC/TERT1, HRTPT, and HREC24T cells, as well as a close association between HREC24T and HK-2 cells (Figure 8A). Flow cytometry analysis confirmed low expression of PROM1 (CD133) in both HK-2 and HREC24T cells (Figure 8B) and high expression of CD24 (Figure 8C), indicating unique transcriptional profiles that differentiate the HRTPT (CD133+CD24+) from HREC24T (CD133-CD24+) and HK-2 cells, and the close association between HREC24T and HK-2 cells.

Given that HREC24T and HK-2 cells share the characteristics of low PROM1 (CD133) expression and high CD24 expression, we extended our transcriptomic analysis to determine whether the response of HK-2 cells under hypoxia would be similar to that of HREC24T cells (i.e., reduced cell viability and decreased EGFR expression). HK-2 cells cultured under hypoxic conditions (2.5% oxygen) for 48 h exhibited a significant 50% reduction in cell viability (Figure 8D) and morphological changes indicative of cell death (Figure 8E). Protein analysis demonstrated that culturing HK-2 cells under hypoxia significantly increased HIF1A protein levels three-fold (Figure 8F), a level comparable to that observed in HREC24T cells under hypoxic conditions. Furthermore, hypoxia significantly reduced EGFR and pEGFR protein levels in HK-2 cells (Figure 8G,H). These results were consistent with those in HREC24T cells.

**Figure 9:**
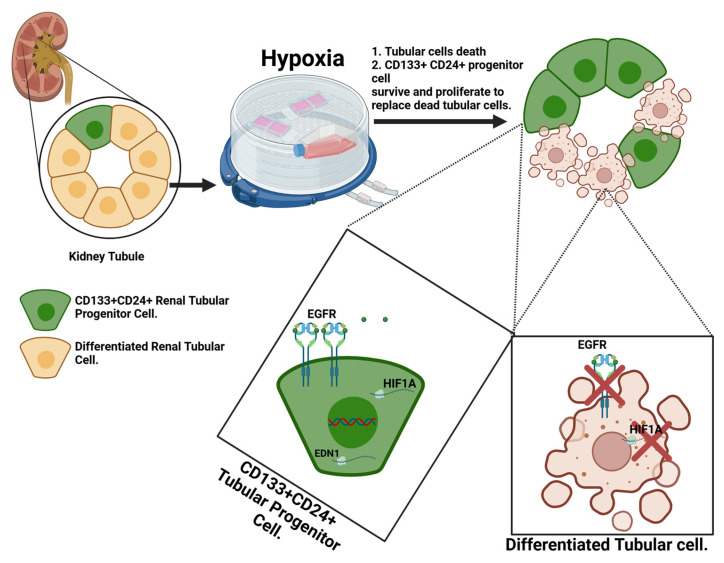
A model illustrating the effects of hypoxia on CD133+CD24+ renal progenitor cells. Differentiated tubular cells fail to upregulate HIF1A and EGFR, leading to hypoxia-induced cell death. In contrast, CD133+CD24+ cells survive the hypoxic injury by upregulating HIF1A and EGFR.

Overall, our results suggest that after hypoxic injury, differentiated tubular cells undergo cell death. In contrast, the ability of CD133+CD24+ cells to upregulate HIF1A and EGFR could contribute to the survival of these cells, followed by cell proliferation to replace the dead tubular cells (Figure 9).

## 3. Discussion

The intrinsic ability of the kidney to repair itself after acute injury presents a unique opportunity to explore the mechanism of this process and harness its potential to promote renal healing. CD133+CD24+ renal progenitor cells play a critical role in repairing tubular damage after injury [7,17,18]. Gaining a deeper understanding of the biology and the repair mechanism of these kidney progenitor cells could provide valuable insights into the kidney’s restorative processes.

Recent advancements in the treatment of kidney injury have shown the potential of using stem cells and extracellular vesicles (EVs) derived from stem cells as frontier therapies, offering regenerative promise where traditional approaches fail. Mesenchymal stem cells (MSCs) have demonstrated renoprotective effects by modulating inflammation, promoting tubular regeneration, and reducing fibrosis in animals with acute kidney injury [19,20,21]. In phase-1 clinical trials, autologous MSC infusion increases blood flow and glomerular filtration rate and attenuates inflammatory injury in post-stenotic kidneys [22]. An alternative to MSC therapy is extracellular vesicles (EVs) derived from stem cells or kidney progenitors, which can enhance kidney repair processes, mitigate oxidative stress, and enhance cell-to-cell communication in damaged renal tissue [23,24,25]. EV-based therapies may even overcome some limitations of direct stem cell transplantation, such as immune rejection or poor engraftment, with preclinical trials showing improved glomerular filtration rates in AKI rodents [26]. However, challenges remain, including optimizing delivery methods, ensuring scalable production, and translating these findings to human clinical outcomes. Together, these approaches represent a cutting-edge convergence of regenerative medicine and nephrology to redefine kidney injury management and create urgency to understand the biology of kidney repair and the effects of kidney stem cells and progenitors in the repair process.

Our laboratory has successfully established and characterized immortalized CD133+CD24+ (HRTPT) renal tubular progenitor cells and CD133-CD24+ (HREC24T) cells, which lack progenitor characteristics [10,13,16]. HRTPT cells provide an unlimited and consistent supply of kidney progenitor cells, allowing comprehensive studies to be performed to understand the nature and biology of the rare, difficult-to-isolate primary CD133+CD24+ kidney progenitor cells.

In this study, we performed a transcriptomic analysis of two distinct cell populations: the HRTPT (CD133+CD24+) and HREC24T (CD133-CD24+) cell lines, which were isolated by fluorescence-activated cell sorting from the parent RPTEC/TERT1 cell line. We identified their differential response to hypoxic stress. Our analysis revealed significant differences in functional pathway enrichment between these two cell populations, highlighting their distinct biological roles. In vitro, we demonstrated a differential response to hypoxia between HRTPT and HREC24T cells and showed the resilience of CD133+CD24+ progenitor cells under hypoxic conditions. This study represents the first comprehensive analysis of the differential effects of hypoxia in immortalized CD133+CD24+ kidney cells versus CD133-CD24+ cells, which identified upregulation of EGFR and HIF1A in CD133+CD24+ progenitor cells under hypoxic conditions, suggesting the potential contribution of these pathways to the resistance of CD133+CD24+ cells to hypoxia.

Canonical pathway analysis revealed that, compared to HREC24T cells, the HRTPT cells are enriched with genes associated with key pathways critical for the pluripotency of human and mouse embryonic stem cells (e.g., *ACVRL1*, *AKT3*, *FZD10*, *ID1*, *ID3*, *ID4*, *JAK3*, *PIK3CD*, *POU5F1*, *PRKCB*, *TGFB2*, *WNT2B*, and *WNT7B*). Furthermore, these cells were enriched with transcriptional regulatory networks that are essential for the functionality of embryonic stem cells, including the *AKT3*, *FZD10*, *HNF4A*, *JAK3*, *PIK3CD*, *POU5F1*, *TGFB2*, *WNT2B*, and *WNT7B* genes. These findings support our earlier conclusions regarding the progenitor characteristics of HRTPT cells [16].

Our Ingenuity Pathway Analysis (IPA) demonstrated that HRTPT cells are enriched in the HIF1A and epidermal growth factor (EGF) signaling pathways. Our in vitro data indicated that under hypoxia, HRTPT cells exhibited a 100-fold increase in HIF1A protein levels, accompanied by an upregulation of EGFR protein. In contrast, HREC24T cells showed only a twofold increase in HIF1A and failed to upregulate EGFR expression, highlighting a striking differential hypoxia response between HRTPT and HREC24T cells.

Hypoxia-inducible factor 1-alpha (HIF1A) is a critical transcription factor that regulates the cellular response to low oxygen levels after being stabilized and translocated to the nucleus, where HIF1A binds to hypoxia-responsive elements (HREs) in the promoter of the target genes to modify the expression profile of the cell to favor pro-survival signaling [17]. Studies have shown that HIF1A protects proximal tubular cells from injury and promotes cell survival and proliferation, while HIF1A knockdown exacerbates tubular cell death [18,27,28]. Our results demonstrated a 100-fold increase in HIF1A expression in HRTPT cells compared to HREC24T cells. HIF1A significant upregulation may explain the minimal impact of hypoxia on cell viability in HRTPT cells compared to HREC24T cells, which demonstrated a 50% reduction in viability and only a two-fold increase in HIF1A expression under hypoxic conditions.

Collectively, these findings suggest that HRTPT (CD133+CD24+) cells in the kidney demonstrate increased resistance to hypoxic injuries and have a robust ability to upregulate the protective HIF1A protein. This upregulation likely contributes to their survival mechanism under hypoxic conditions and enhances their ability to proliferate and replace the damaged tubular cells. Studies conducted on primary CD133+ kidney progenitor cells showed a correlation between HIF1A and CD133 expressions, with hypoxia shown to increase the expression of stem cell markers and clonogenicity [29]. However, knockdown of HIF1A in CD133+ cells will be required to determine if HIF1A is the sole signal responsible for the survival of CD133+ progenitors under hypoxia.

Epidermal growth factor (EGF) and its receptor (EGFR) have been recognized as a double-edged sword in kidney injury [30]. Studies indicate that the EGFR-signaling pathway is essential for the repair of damaged tubules after the initial phase of ischemic injury and plays a crucial role in stimulating proximal tubular cell proliferation. Moreover, mice with EGFR point mutations exhibit slower recovery from ischemic reperfusion injury in the kidney [31]. Furthermore, EGFR knockdown or pharmacological inhibition impairs kidney repair after injury, while EGF supplementation accelerates the repair process [32,33]. Activation of the EGFR signaling pathway is vital for renal tubular cell survival and proliferation after injury [34]; however, persistent activation of EGFR has been implicated in the pathogenesis of kidney fibrosis, which is a hallmark of chronic kidney disease [35,36,37,38]. Sustained or aberrant activation of EGFR can drive pathological processes, including epithelial-to-mesenchymal transition (EMT), inflammation, and extracellular matrix (ECM) deposition. These processes collectively contribute to fibrosis by promoting myofibroblast activation and excessive accumulation of ECM, leading to structural damage and loss of renal function. Emerging evidence suggests that EGFR activation in response to kidney injury enhances the production of profibrotic mediators, such as transforming growth factor-beta (TGF-β) and connective tissue growth factor (CTGF), which amplify fibrotic signaling cascades [34,35,37,39,40]. Furthermore, studies in preclinical models of chronic kidney disease demonstrate that pharmacological inhibition of EGFR attenuates fibrosis, highlighting its potential as a therapeutic target [41]. Understanding the role of EGFR in the kidney repair process and progression to fibrosis provides critical insights into the progression of CKD and identifies potential avenues for intervention [37]. Our results showed that in short-term hypoxia, HRTPT progenitor cells upregulated EGFR and pEGFR proteins; in contrast, HREC24T cells failed to upregulate EGFR and pEGFR. This could be another factor contributing to the survival of progenitor HRTPT cells under hypoxia compared to HREC24T cells that did not upregulate EGFR under hypoxia and showed cell death. However, it is necessary to investigate whether the upregulation of EGFR in CD133+CD24+ is a transient or persistent phenomenon to identify if CD133+CD24+ cells are a source of persistent activation of EGFR, which could participate in future kidney fibrosis after injury.

Our functional network analysis of differentially expressed genes in HRTPT cells revealed a significant connection between epidermal growth factor (EGF) and hypoxia-inducible factor 1-alpha (HIF1A) through a central node representing the Endothelin-1 (*EDN1*) gene. Endothelin-1 (ET-1) is a potent vasoconstrictor peptide synthesized by endothelial cells, mesangial cells, and tubular epithelial cells, which plays a crucial role in regulating renal function [42]. Specifically, ET-1 modulates renal blood flow, glomerular filtration rate, and excretion of sodium and water. ET-1 exerts its effects through endothelin receptors located in various renal structures. Dysregulation of ET-1 activity has been implicated in numerous renal diseases, including chronic kidney disease, leading to increased vascular resistance, inflammation, and fibrosis [34,35]. Importantly, the *EDN1* gene contains a hypoxia response element within its promoter region, making it a target for HIF1A-mediated transcriptional regulation [36]. This could explain the upregulation in HRTPT cells but not HREC24T cells, as HRTPT cells upregulated HIF1A 100-fold more than HREC24T cells. Furthermore, EGFR signaling has been shown to enhance EDN1 transcription through multiple downstream pathways, including MAPK/ERK, PI3K/AKT, JAK/STAT, and PKC [34,37]. Our findings indicate that hypoxia significantly upregulates EDN1 in HRTPT cells, suggesting that CD133+CD24+ progenitor cells may be a key source of endothelin-1 upregulation after hypoxic kidney injury. However, CD133 knockdown and overexpression studies are recommended to determine the connection between CD133 and EDN1, which could provide another future approach for targeting EDN1.

Our results demonstrated that the response of HREC24T cells to hypoxia is comparable to the response of the well-characterized HK-2 proximal tubular cell line. Under hypoxia, both HREC24T and HK-2 cells exhibited decreased cell viability and failed to upregulate EGFR protein levels. Additionally, both cell lines displayed only a modest increase in HIF1A protein levels, in stark contrast to the hundred-fold increase that was observed in HRTPT progenitor cells. Notably, neither HREC24T nor HK-2 cells expressed CD133, reinforcing their non-progenitor characteristics and indicating the role of CD133 in the resistance to hypoxic stress. Previous studies have shown that primary CD133+ kidney cells are resistant to cell death induced by hemoglobin and nephrotoxic agents [7,43], which corroborates our findings in HRTPT cells. Future studies are needed to determine if overexpression of CD133 in HK-2 and HREC24T cells will increase their resistance to hypoxia.

Our results indicate the importance of the CD133, EGFR, and HIF1A pathways in the survival of immortalized CD133+CD24+ tubular cells following acute hypoxic injury. Specifically, our data suggest that targeting these pathways could help mitigate tubular cell death and promote regeneration after acute kidney damage. Repurposing existing drugs or developing new therapeutics that activate or modulate the CD133, EGFR, and HIF1A may offer promising treatment strategies for hypoxia-induced kidney injury. Our results pave the way for translational research aimed at developing targeted interventions to improve clinical outcomes in patients with acute kidney injuries.

## 4. Limitations of the Study

The lack of in vivo and clinical data limits this study; however, the results lay the ground for future animal studies. Although glycosylated CD133 is not detectable in mice, other markers of murine kidney progenitors should be used.

## 5. Conclusions

Our findings indicate that CD133+CD24+ renal tubular progenitor cells have greater resistance to hypoxic injury compared to CD133-CD24+ renal tubular cells. CD133+CD24+ cells could be the source of increased expressions of HIF1A, EGFR, and EDN1 after injury, which could explain the ability of CD133+CD24+ renal progenitor cells to survive hypoxic kidney injury and subsequently proliferate to repair and replace damaged tubular cells. Although these results are promising, further functional validation is necessary. As a future direction, we recommend experimental knockdown of CD133 and EGFR in CD133+CD24+ renal tubular progenitor cells to better understand how these pathways influence cell survival, proliferation, and response to hypoxia and nephrotoxic stress. Additionally, we recommend investigating the possible impact of prolonged hypoxia on these progenitor cells and their role in fibroblast activation. These studies will provide crucial insights into the interplay of these signaling pathways and their implications for kidney injury and repair.

## 6. Methods

### 6.1. Cell Culture and Reagents

RPTEC/TERT1 and HK-2 cells were acquired from the American Type Culture Collection (ATCC). HRTPT and HREC24T cell lines were previously established in our laboratory [10,15]. All cells were cultured under serum-free conditions using a 1:1 mixture of Dulbecco’s Modified Eagle’s Medium (DMEM) and F-12 serum-free medium (Thermo Fisher Scientific, Waltham, MA, USA). The culture medium was supplemented with selenium (5 ng/mL), insulin (5 μg/mL), transferrin (5 μg/mL), hydrocortisone (36 ng/mL), triiodothyronine (4 pg/mL), and epidermal growth factor (10 ng/mL). Cells were maintained at 37 °C in a 5% CO_2_ atmosphere, with the medium being replaced every two days. Upon reaching confluency, the cells were detached using the TrypLE enzyme (#12563029, Thermo Fisher Scientific) and passaged at a 1:4 ratio.

### 6.2. Flow Cytometry Analysis

Cells were seeded in a 6-well plate at a density of 250,000 cells per well and allowed to grow until confluency, indicated by dome formation. Cells were detached using the TrypLE enzyme and then centrifuged at 1200 rpm for three minutes. Cells were subsequently transferred to new Falcon™ tubes (#352235, Thermo Fisher Scientific) and resuspended in a staining buffer composed of phosphate-buffered saline (PBS) supplemented with 5% bovine serum albumin. Cells were labeled with APC-conjugated anti-human PROM1 (AC133) primary antibody (#130-113-106, Miltenyi Biotec, Bergisch Gladbach, Germany) and FITC-conjugated anti-human CD24 primary antibody (#130-127-493, Miltenyi Biotec, Bergisch Gladbach, Germany) for 30 min in dark followed by five minutes wash in PBS for three times. Flow cytometry and cell sorting were conducted using a Sony SH800S cell sorter, and the results were analyzed with FlowJo™ v10.8 software (BD Life Sciences, Franklin Lakes, NJ, USA).

### 6.3. RNA Extraction and RT-qPCR

Cell pellets were collected by cell scraping in sterile PBS and were rapidly frozen using liquid nitrogen. Cell pellets were lysed with 350 μL of the proprietary RLT^®^ buffer (Qiagen, Hilden, Germany), then dissociated using QIAshredder tubes (Qiagen) and centrifuged at 12,500 rpm for two minutes. RNA was isolated using the RNeasy Mini Plus Kit (#74034, Qiagen) and the QIAcube instrument (Qiagen) in accordance with Qiagen’s established protocol. RNA quantity was measured using a NanoDrop spectrophotometer (Thermo Fisher Scientific), while RNA quality, including the RNA Integrity Number (RIN), was assessed with Agilent 4200 TapeStation (Agilent Technologies, Santa Clara, CA, USA). A total of 1 µg of complementary DNA (cDNA) was synthesized from the purified RNA using the LunaScript^®^ RT SuperMix Kit (#E3010L, New England Biolabs, Ipswich, MA, USA). The cDNA was diluted with nuclease-free water to achieve a final concentration of 10 ng/µL. For quantitative PCR (qPCR), 20 ng of cDNA was used in a 20 µL reaction. The analysis was conducted using the BioRad CFX96 Touch Real-Time PCR thermocycler (BioRad, Hercules, CA, USA) and the Luna^®^ Universal qPCR Master Mix (#M3003E, New England Biolabs). The qPCR conditions were as follows: one cycle at 95 °C for 2 min, followed by 40 cycles consisting of 5 s at 95 °C and 30 s at 60 °C. Expression levels were determined using threshold cycle values (Cq) and the 2^−∆∆Ct^ method, with 18S serving as the reference gene. Primers were purchased from Integrated DNA Technologies (IDT) in Coralville, IA, USA, and the sequences are provided in the Supplemental File (Table S5).

### 6.4. RNA Sequencing and Analysis

For RNA sequencing, total RNA was extracted from cells using a Qiagen RNA isolation kit to ensure high purity and integrity. RNA samples were assessed for quality using a bioanalyzer, and libraries were prepared using a strand-specific RNA sequencing protocol to capture both coding and non-coding RNAs. Sequencing was performed on an Illumina platform using the Illumina TruSeq Total RNA kit and modified Illumina TruSeq PCR reaction with KAPA HiFi HotStart Ready Mix for the final PCR step to fit the Aviti workflow. Libraries were sequenced to ~40 M 75 bp paired-end reads on the Element Aviti, generating paired-end reads. The raw reads were quality-checked and trimmed to remove adapters and low-quality bases. Subsequently, high-quality reads were aligned to the human reference genome (GRCh38) using HISAT2, and transcript abundance was quantified with FeatureCounts. Differential gene expression analysis was performed using DESeq2, to compare the gene expression profiles of RPTEC/TERT1, HRTPT, and HREC24T cells. Genes with *p*-values < 0.05 were considered statistically significant. To further refine the list of DEGs, we applied a fold-change cutoff of ≥2.0 (upregulated or downregulated). This stringent threshold allowed us to focus on genes with substantial expression changes that are most likely to contribute to the functional and phenotypic differences between kidney progenitor and non-progenitor cells. Further, this analysis provided insight into the molecular differences between progenitor and non-progenitor kidney cells, revealing key regulatory pathways involved in their distinct functions.

Pathway enrichment analyses were conducted using Ingenuity Pathway Analysis (IPA) to uncover the biological processes and signaling pathways enriched in HRTPT kidney progenitor cells. This analytical approach facilitated the identification of key functional categories associated with the differentially expressed genes (DEGs), allowing for a comprehensive understanding of the molecular mechanisms underpinning the unique characteristics of HRTPT cells. By integrating the gene expression data with IPA’s curated databases, we were able to discern significant biological functions, cellular processes, and signaling pathways that are upregulated in HRTPT progenitor cells.

### 6.5. Hypoxic Cell Culture

According to the experimental protocol, cells were seeded into 10 cm dishes or 12-well plates. After reaching confluency, the culture medium was refreshed, and the cells were incubated under hypoxic conditions (2.5% oxygen) for 24, 48, and 72 h using a hypoxia chamber (#27310, STEMCELL Technologies, Vancouver, BC, Canada). Cells cultured under atmospheric oxygen levels served as the control group. Cells were washed with phosphate-buffered saline (PBS), scraped using a cell scraper, and collected into 2 mL Eppendorf tubes. The samples were then centrifuged at 3000 rpm at 4 °C, after which the supernatant was removed. The cell pellets were rapidly frozen in liquid nitrogen and stored at −80 °C for subsequent RNA or protein analysis.

### 6.6. Crystal Violet Cell Viability

Cells cultured in a 12-well plate were incubated with 0.25% crystal violet solution containing 20% methanol for 15 min. Excess crystal violet was removed by rinsing with double-distilled water, and plates were allowed to air-dry overnight. The cells were lysed with 0.1 M sodium citrate in 25% ethanol (pH 4.2), and the absorbance at 570 nm was measured using a microplate spectrophotometer (BioTek EL800, Santa Clara, CA, USA).

### 6.7. Protein Analysis

Simple Western™ blotting was used to quantify protein expression, as previously described [44]. In brief, cells were washed with ice-chilled phosphate-buffered saline (PBS) and then lysed in radio-immunoprecipitation assay (RIPA) buffer supplemented with 1 mM PMSF, a protease inhibitor cocktail, and sodium orthovanadate (Santa Cruz Biotechnology, Dallas, TX, USA). The cell lysate was subjected to two rounds of sonication, each lasting 15 s, and then centrifuged at 12,000× *g* for 15 min. Protein concentration was determined using the Pierce Bicinchoninic Acid (BCA) Protein Assay Kit (Thermo Scientific Pierce, Waltham, MA, USA). A standard molecular weight kit was used to prepare the samples, in which protein lysates were combined with a 5× fluorescent master mix (ProteinSimple™, San Jose, CA, USA) containing dithiothreitol, fluorescent standards, and a system control protein (26 kDa), followed by heating at 95 °C for five minutes to denature the protein lysate. Samples were loaded into the loading plates per the manufacturer’s protocol, and the protein lysate was separated and immunodetected using a capillary-based Jess Simple Western™ instrument (ProteinSimple™, San Jose, CA, USA), according to the manufacturer’s instructions. The ProteinSimple™ Jess system utilized a 26 kDa protein as an internal control to normalize protein expression across different samples. The antibodies that were used, dilutions, and loaded protein concentrations are listed in the Supplemental File (Table S6). Uncropped blot images are included in the Supplemental File (Figures S1 and S2).

### 6.8. Statistical Analysis

RNA-seq raw reads were stored in FASTQ format files, containing sequence data. Quality assessment of the raw sequencing data was conducted using FastQC (version 0.12.1; Babraham Research Campus, Cambridge, UK) to evaluate sequence quality metrics such as base quality scores, GC content, and adapter contamination. Based on the quality control report, pre-processing of the data was performed using fastp (version 0.17.0; Shenzhen Institutes of Advanced Technology, Chinese Academy of Sciences, Nanshan District, Shenzhen, China), which efficiently trimmed low-quality bases and removed adapter sequences to improve downstream analysis reliability.

Post-pre-processing, STAR aligner (version 2.7.10b; Cold Spring Harbor Laboratory and Pacific Biosciences, Woodbury, NY, USA) was used to align the cleaned reads to the reference genome, ensuring high mapping accuracy and performance. From the aligned reads, a count matrix was generated using FeatureCounts (version 2.0.7; The University of Melbourne, Parkville, Australia), which assigns reads to genomic features to quantify gene expression.

For normalization and differential expression analysis, the DESeq2 package (version 1.46.0; European Molecular Biology Laboratory, Heidelberg, Germany) was utilized. Counts were normalized using the median-of-ratios method to account for sequencing depth and compositional biases across samples. To identify statistically significant differentially expressed genes, a two-tailed Student’s *t*-test was applied with a significance threshold of *p* < 0.05. Principal component analysis (PCA) was performed to visualize sample distributions and evaluate global gene expression variability across conditions in a two-dimensional space. Additionally, Pearson correlation analysis was carried out to assess relationships between samples and genes under different passage conditions, with results visualized using a heatmap to identify the most correlated groups.

**Figure 10:**
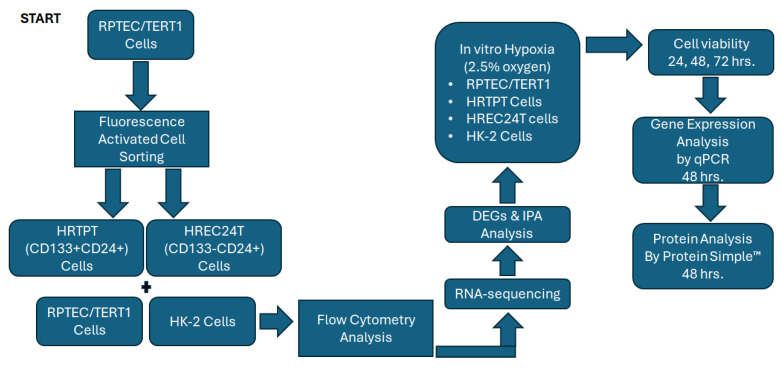
Experimental Design. A diagram showing the sequence of the experimental work and methodology.

The entire RNA-seq pipeline was implemented in R (version 4.3.2; R Foundation for Statistical Computing, Vienna, Austria) and Bioconductor (version 3.20), ensuring a reproducible analysis framework. Experiments were conducted using a minimum of three biological replicates (*n* = 3), and statistical significance was rigorously assessed. The flow of the experimental design is summarized below (Figure 10).

## Data Availability

https://www.ncbi.nlm.nih.gov/geo/query/acc.cgi?acc=GSE280279.

## Supplementary Materials

The following supporting information can be downloaded at: https://www.mdpi.com/article/10.3390/ijms26062472/s1.

## Abbreviations

HRTPT	Human renal tubular precursor TERT.
HREC24T	Human renal epithelial cell 24 TERT.
RPTEC/TERT1	Renal proximal tubular epithelial cells-TERT1.
EGFR	Epidermal growth factor receptor
EDN1	Endothelin-1
ET-1	Endothelin-1 protein.
HIF1A	Hypoxia-Inducible Factor 1A.
