# Supplementary material for: CD133+CD24+ Renal Tubular Progenitor Cells Drive Hypoxic Injury Recovery via Hypoxia-Inducible Factor-1A and Epidermal Growth Factor Receptor Expression
Source: Int J Mol Sci. 2025 Mar 10;26(6):2472. doi: 10.3390/ijms26062472 (PMC11942380; doi:10.3390/ijms26062472)
Supplement: Supplementary file 1 [file ijms-26-02472-s001.zip › Figure S1.pdf]

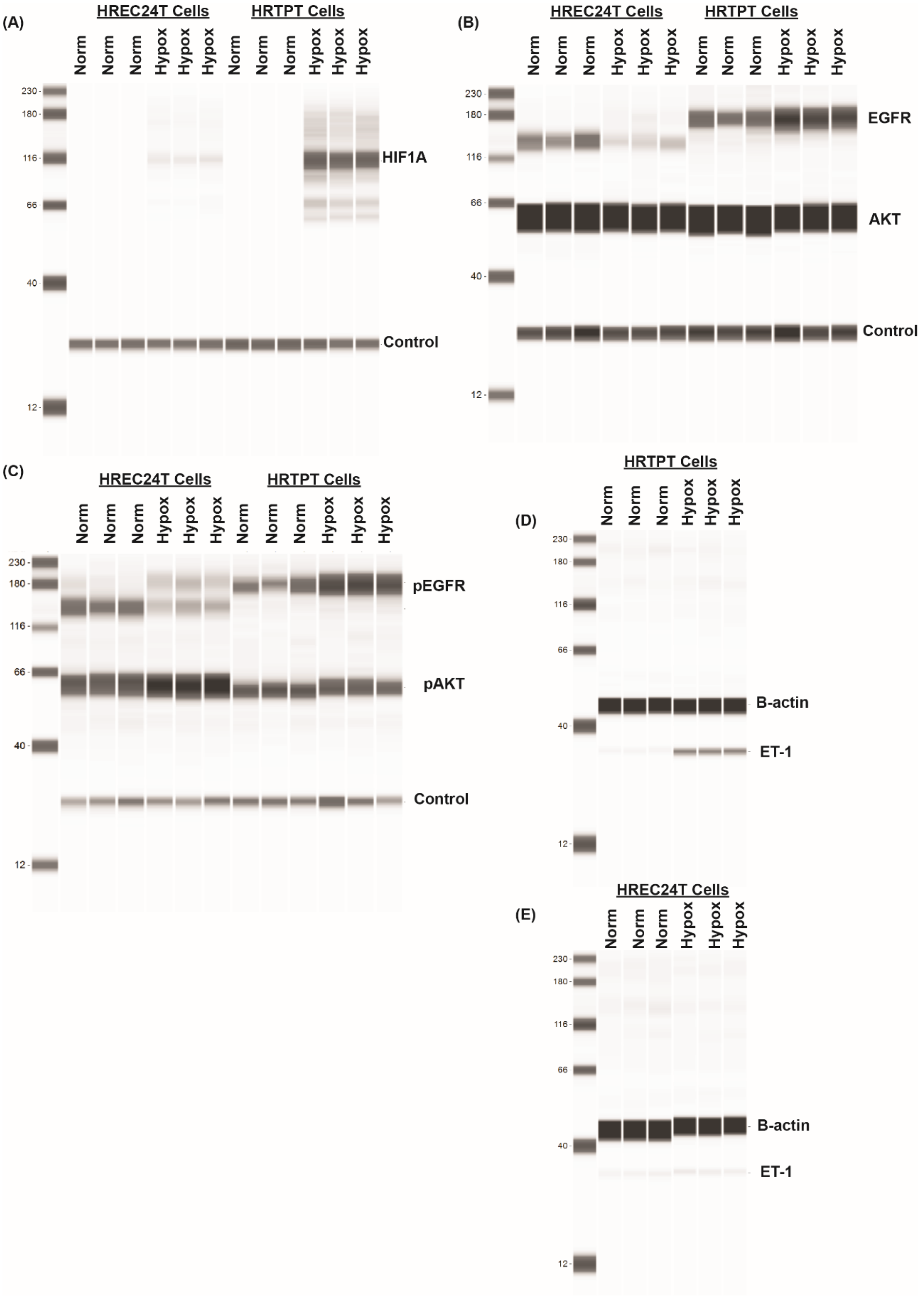

Figure S1: Full, uncropped Simple Western blots for the protein bands shown in Figure 7 of the manuscript, corresponding to the following proteins: (A) HIF1A, (B) EGFR, (C) pEGFR, and (D, E) endothelin-1 (ET-1) in HRTPT and HREC24T cells.
