# Supplementary material for: CD133+CD24+ Renal Tubular Progenitor Cells Drive Hypoxic Injury Recovery via Hypoxia-Inducible Factor-1A and Epidermal Growth Factor Receptor Expression
Source: Int J Mol Sci. 2025 Mar 10;26(6):2472. doi: 10.3390/ijms26062472 (PMC11942380; doi:10.3390/ijms26062472)
Supplement: Supplementary file 1 [file ijms-26-02472-s001.zip › Table S5.pdf]

Table S5: Primers used in gene expression analysis.

Gene	Catalog No./unique Assay ID	Source
EDN1	Hs.PT.58.1079817	Integrated DNA Technologies
FN1	Hs.PT.58.40005963	Integrated DNA Technologies
TGFA	Hs.PT.58.22647128	Integrated DNA Technologies
VEGFA	Hs.PT.58.1149801	Integrated DNA Technologies
18S	5’-CGCCGCTAGAGGTGAAATTC-3’<br>5’-TTGGCAAATGCTTTCGCTC-3’	Integrated DNA Technologies
RPLP0	Hs.PT.39a.22214824	Integrated DNA Technologies
