# Supplementary material for: CD133+CD24+ Renal Tubular Progenitor Cells Drive Hypoxic Injury Recovery via Hypoxia-Inducible Factor-1A and Epidermal Growth Factor Receptor Expression
Source: Int J Mol Sci. 2025 Mar 10;26(6):2472. doi: 10.3390/ijms26062472 (PMC11942380; doi:10.3390/ijms26062472)
Supplement: Supplementary file 1 [file ijms-26-02472-s001.zip › Table S6.pdf]

Table S6: Antibodies used in SimpleWestern™ and Flow cytometry analysis.

Protein	Antibody	Dilution	Host	Protein loading Conc
HIF1A	Cell Signaling Cat#36169	1:200	Rabbit	0.25µg/µl.
EGFR	Cell Signaling Cat#4267	1:300	Rabbit	0.5µg/µl.
pEGFR	Cell Signaling Cat#3777	1:300	Rabbit	0.5µg/µl.
Endothelin 1 (ET-1)	Sanat Cruz Biotechnology Cat#sc-517436	1:50	Mouse	1µg/µl.
AKT	Cell Signaling Cat#4060S	1:100	Rabbit	0.5µg/µl.
pAKT	Cell Signaling Cat#9272S	1:100	Rabbit	0.5µg/µl.
β-actin	Cell Signaling Cat#3700S	1:200	Mouse	0.5µg/µl.
APC-CD133	Miltenyi Biotech Cat#130-113-106	1:10	Mouse	N/A
FITC-CD24	Miltenyi Biotech Cat#130-127-493	1:10	Mouse	N/A
